# Dopamine and Levodopa Prodrugs for the Treatment of Parkinson’s Disease

**DOI:** 10.3390/molecules23010040

**Published:** 2017-12-25

**Authors:** Fatma Haddad, Maryam Sawalha, Yahya Khawaja, Anas Najjar, Rafik Karaman

**Affiliations:** Department of Bioorganic & Pharmaceutical Chemistry, Faculty of Pharmacy, Al-Quds University, Jerusalem P.O. Box 20002, Palestine; iamfromhebron@hotmail.com (F.H.); maryam_khaled2@yahoo.com (M.S.); yahya.khawaja@hotmail.com (Y.K.); nash.najjar@gmail.com (A.N.)

**Keywords:** dopamine, levodopa, prodrug, Parkinson’s disease, blood brain barrier

## Abstract

*Background*: Parkinson’s disease is an aggressive and progressive neurodegenerative disorder that depletes dopamine (DA) in the central nervous system. Dopamine replacement therapy, mainly through actual dopamine and its original prodrug l-dopa (LD), faces many challenges such as poor blood brain barrier penetration and decreased response to therapy with time. *Methods*: The prodrugs described herein are ester, amide, dimeric amide, carrier-mediated, peptide transport-mediated, cyclic, chemical delivery systems and enzyme-models prodrugs designed and made by chemical means, and their bioavailability was studied in animals. *Results:* A promising ester prodrug for intranasal delivery has been developed. LD methyl ester is currently in Phase III clinical trials. A series of amide prodrugs were synthesized with better stability than ester prodrugs. Both amide and dimeric amide prodrugs offer enhanced blood brain barrier (BBB) penetration and better pharmacokinetics. Attaching LD to sugars has been used to exploit glucose transport mechanisms into the brain. *Conclusions*: Till now, no DA prodrug has reached the pharmaceutical market, nevertheless, the future of utilizing prodrugs for the treatment of PD seems to be bright. For instance, LD ester prodrugs have demonstrated an adequate intranasal delivery of LD, thus enabling the absorption of therapeutic agents to the brain. Most of the amide, cyclic, peptidyl or chemical delivery systems of DA prodrugs demonstrated enhanced pharmacokinetic properties.

## 1. Introduction

**Scheme 1:**
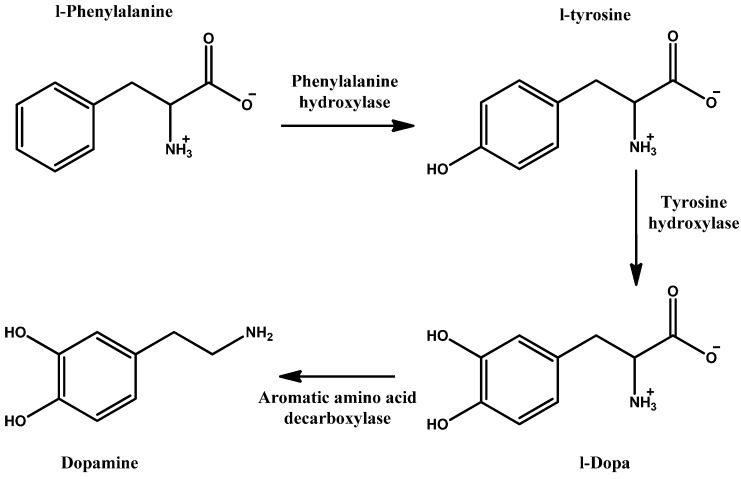
Dopamine synthesis pathway.

**Figure 1:**
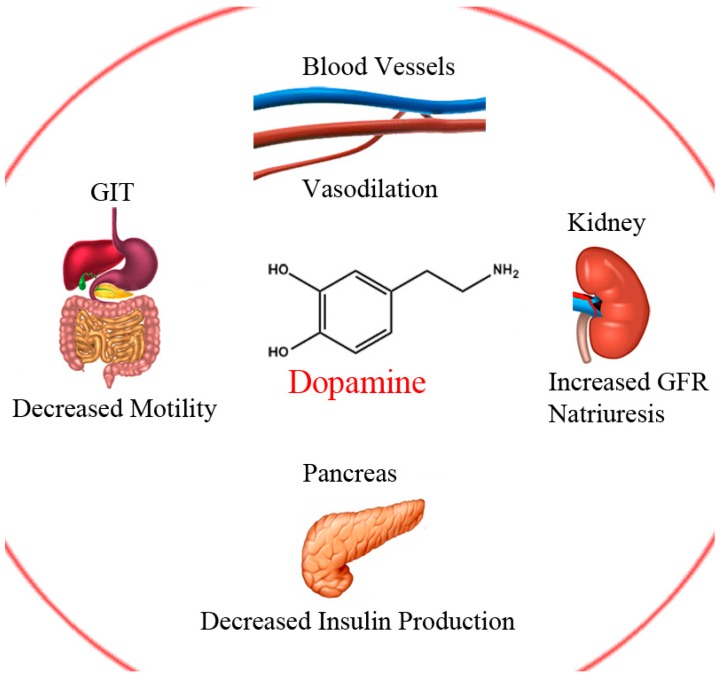
Select peripheral actions of dopamine.

Dopamine (DA) is a natural neurotransmitter and neurohormone that exerts its action by binding to five DA receptors in the brain, D_1–5_ [1]. DA production represents the first steps in catecholamine synthesis and starts with phenylalanine which is hydroxylated to tyrosine, then again to levodopa (LD) and finally, through decarboxylation, to DA (Scheme 1). This cascade is accomplished and catalysed with the aid of three enzymes. The rate limiting enzyme is tyrosine hydroxylase. This can be inhibited by catecholamine neurotransmitters through negative feedback to keep proper regulation of DA [2]. DA plays several and variant roles in the body systems; including central nervous system (CNS), circulatory, renal, digestive and immune systems (Figure 1). Its role in the CNS is a cornerstone in controlling movement and emotion [1].

Parkinson’s disease (PD) is the second most common neurodegenerative disorder, affecting about 1% of persons over the age of 60, causing progressive neurodegenerative movement disorders [3], along with cognitive manifestations; making the patient neurologically disabled. PD has global distribution with no gender preference [4,5]. The pathological explanation of PD symptoms is mainly due to the diminished dopamine in the basal ganglia. In the healthy brain, exactly in the midbrain region, there is an area named substantia nigra where the degeneration of neuronal cells happens. This area communicates with the striatum using the chemical messenger DA. Whereas, in the diseased brain, these cells are depleted, hence, the DA deficits in striatal cells. The other major neuropathological symptom of PD is the existence of Lewy bodies and Lewy neuritis [6]. The exact cause of neuronal deathis still not completely understood but might be due to proteasomal as well as mitochondrial dysfunction [4,7].

Pharmacologic treatment of PD is mainly symptomatic based on DA replacement therapy, as exogenous DA and other catecholamines cannot be administered due to their poor brain blood barrier (BBB) penetration [1]. There are a number of drugs available for the treatment of PD. These include agonists of dopamine (rotigotine, ropinirole, and lisuride), cholinesterase inhibitors (donepezil), antimuscarinic drugs (trihexyphenidyl, benztropine, biperiden), MAO-B inhibitors (selegiline, rasagiline), amantadine, and other DA and LD prodrugs [8,9].

However, administering external dopamine to PD patients as treatment is limited, as dopamine is a water-soluble hydrophilic drug that does not satisfy the characteristics of a substance that can enter the brain by BBB penetration [1,10,11].

This penetration can be achieved by one of three main ways. First, a drug has to diffuse freely through the membrane if it obeys Lipinski’s rule of five, suggested and applied since 1997 [12], with minor exceptions. Secondly, one which can permeate via active transport through specific carriers such as amino acid and nucleoside carriers. Thirdly, a drug or substance which can be internalised either by electrostatic interactions with specific endothelial membranes (adsorptive-mediated transport) or through receptor mediated endocytosis, as is the case as with proteins and peptides [13,14,15,16].

l-Dopa (LD), is the direct precursor of DA and is a suitable prodrug as it facilitates CNS penetration and delivers DA. It has been, and still is, considerably the golden standard therapy for PD especially in the early stages [17,18,19]. Nevertheless, chronic long-term treatment with LD causes motor complications (on-off phenomenon) in the majority of patients. In addition, dyskinesia may occur due to excess dopaminergic tone [20,21]. In the early stages of PD, LD is a very effective treatment of PD [18,19], particularly when given in combination with carbidopa, a decarboxylase inhibitor. Carbidopa works on the peripheral decarboxylase inhibitor and cannot pass the BBB; its role is to decrease the peripheral breakdown of LD, thus, mostly avoiding the drug’s systemic side effects [22,23]. 

In normal brain tissue the basal ganglia maintains the brain’s needs of DA for motor control and others, but LD oral administration has low bioavailability (10% with only 1% of LD reaching the brain) due to erratic gastrointestinal metabolism of the drug before it attaches to the l-amino acid carrier that transports the drug actively through the duodenum where it enters the bloodstream intact [24,25,26,27,28,29]. Thus, lessened amounts of DA puts the brain under fluctuations that are hard to accommodate [30,31]. Other side effects may occur systemically, including cardiac arrhythmias, hypotension and vomiting as a result of peripheral LD metabolism [22,24]. Strategies to overcome these problems were established to ensure the presence of constant levels of DA in a PD patient’s brain. One strategy was to inhibit LD breakdown in peripheral tissues, consequentially overcoming systemic side effects. Immediate release preparations of LD have a short t_½_ of 1–3 h [25], which can be prolonged by the inhibition of catechol-*O*-methyl transferase (COMT) through entacapone and tolcapone, or dopa-decarboxylase (DDC) through carbidopa. As a result, levels of LD reaching the BBB will be increased, therefore increasing their entrance and maintaining near normal levels of DA in the brain [26,27,30,32].

Therefore, various strategies were applied to conquer CNS penetration. Direct administration of the drug into the brain, temporal interruption of the BBB, addition of chemical groups to the needed substance for enhancing its permeability (the prodrug approach) and formulation of carriers with lipophilic surfaces including liposomes, nanoparticles, dendrimers, as well as micelles [33,34]. In the last few decades, the use of the prodrug approach has gained increased interest. It includes editing the drug’s physicochemical properties, through conjugation with another moiety for example, to overcome pharmacokinetic or pharmacodynamic hurdles, with the intention of releasing the parent drug post activation or degradation of the prodrug [11,35,36,37,38].

In this review, strategies employed in the synthesis of l-dopa and dopamine prodrugs are highlighted based on the type of prodrug synthesized. Those strategies can be classified as: ester, amide, dimeric amide, carrier-mediated, and cyclic prodrugs as well as chemical delivery systems and enzyme models.

### 1.1. Ester Prodrugs

A widely applied and common strategy for prodrug synthesis is the use of esters [39,40,41]. By exploiting ester linkages, which are susceptible to both chemical and enzymatic hydrolysis, molecules can be synthesized with the aim of yielding the active pharmacological compound once the linkage is cleaved.

**Figure 2:**
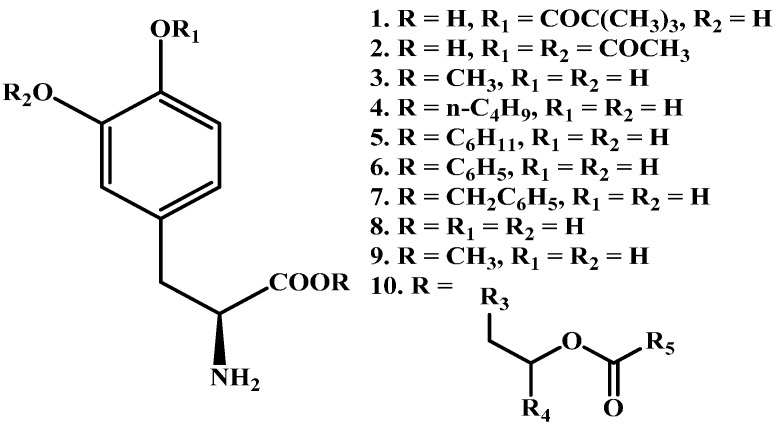
Chemical structures of LD ester prodrugs, **1**–**10**.

A series of promising LD ester prodrugs were previously synthesized in an attempt to increase the bioavailability of LD (**1**–**9** in Figure 2) [42,43,44,45,46,47,48]. However, none of those demonstrated significantly longer duration of action or potency. XP21279 (**10** in Figure 2) was a drug patented by Xenoport (later acquired by Arbor Pharmaceuticals, Atlanta, GA, USA). The drug held potential for sustained release formulations due to lower GI tract absorption. Originally it showed potential to reduce dopamine fluctuations and was expected to decrease “off time” dyskinesia. In a phase II trial [49] investigating XP21279-carbidopa combination, it was found that it could be administered 3 times daily, but failed to show marked reductions in off time.

Etilevodopa (TV 1203), an ethyl ester of LD was produced [50]. The prodrug was rapidly hydrolysed by esterases in the duodenum and absorbed as LD. Etilevodopa appeared to have increased solubility and bioavailability with shorter time to C_max_. However, no marked improvement in on-off fluctuations in PD patients was recorded and hence the prodrug was dropped.

**Figure 3:**
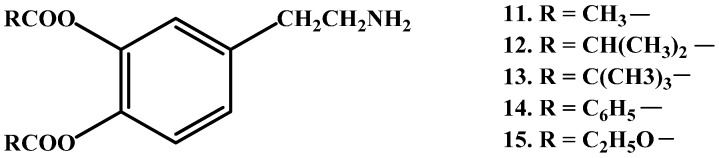
Chemical structures of lipophilic 3,4-*O*-diester prodrugs of DA, **11**–**15**.

Casagrande et al. [51] and Borgman et al. [52] have prepared latentiated lipophilic derivatives of DA. The series of lipophilic 3,4-*O*-diesters (**11**–**15** in Figure 3), are intended for use in the treatment of parkinsonism, hypertension and renal failure. Unfortunately, the results demonstrated that O-acetylation was not sufficient to allow penetration into CNS.

Also, nasal powder preparations of the prodrug, LD methyl ester hydrochloride (LDME) (**3** in Figure 2), hold potential benefit for alternate routes of delivery with bioavailability figures reaching 66.7% and 82.4% with and without Carbopol, respectively [53]. Combining LD methyl ester hydrochloride with trans buccal delivery would be expected to have higher bioavailability than LD and be capable of maintaining better plasma levels of the drug [54]. LDME is currently in Phase III clinical trials and expected to be with decreased adverse effects [55].

### 1.2. Amide Prodrugs

**Figure 4:**
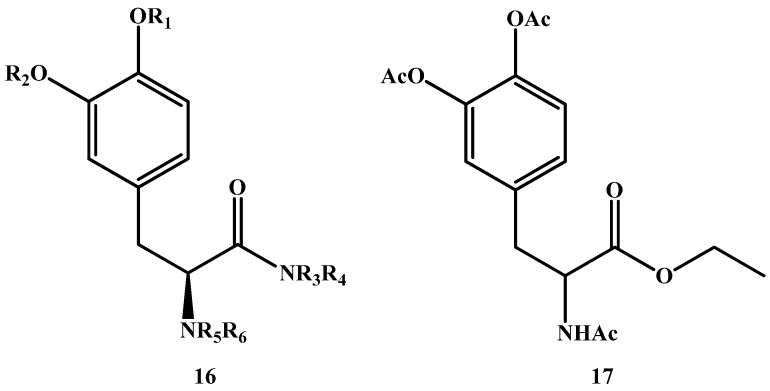
Chemical structures of general amide prodrugs (**16**) and (*S*)-4-(2-acetamido-3-ethoxy-3-oxopropyl)-1,2-phenylene diacetate (**17**).

In addition to the abovementioned ester prodrugs, amide prodrugs are also capable of increasing the lipophilicity of DA and hence, the ability to cross the BBB through passive diffusion, making them a logical option in LD prodrug synthesis. Though, and in contrast to esters, amides are less susceptible to hydrolysis. A general formula for potential amides is depicted in (**16** in Figure 4) [56].

Jiang et al. [57] synthesized and tested an amide prodrug on rats (**17** in Figure 4). In vivo study results showed enzymatic hydrolysis and release of LD after oral administration with C_max_ being 1980.7 ± 538.5 vs. 1936.6 ± 114.6 ng/mL, T_max_ was 24.5 ± 3.5 vs. 4.5 ± 0.8 min, AUC was 217,158.9 ± 70,832.1 vs. 94,469.5 ± 7183.0 ng/mL min and the t_½_ was 56.5 ± 14.4 vs. 30.6 ± 1.6 h for the amide prodrug and l-dopa respectively.

**Figure 5:**
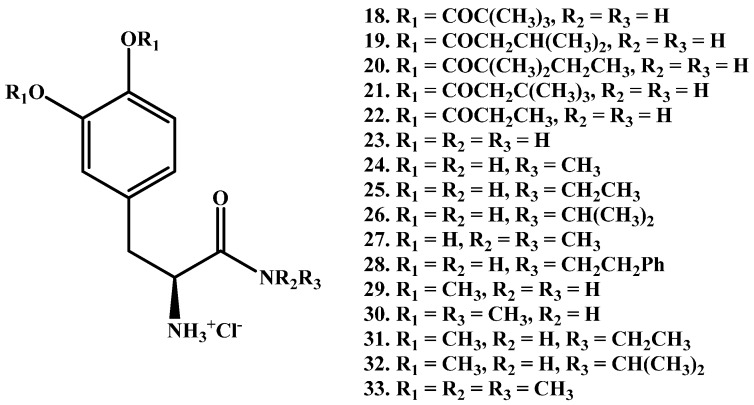
Chemical structures of a synthetic series of amide prodrugs, **18**–**33**.

Zhou et al. [58] reported the synthesis of a series of new LD amides (l-dopamides, **18**–**33** in Figure 5) studied in unilaterally 6-hydroxydopamine (6-OHDA)-lesioned rats. The authors declared that less l-dopa was obtained from compounds with more hydrophobic ester protecting groups (structures **18**–**22**) compared with acetyl compounds (structure **29**). This is due to slower hydrolysis of amides when compared to hydrolysis-susceptible esters which exhibit poor plasma stability. Prodrug **29** showed the best results with prolonged duration of action (147 min) vs. LD (119 min), following oral administration.

**Figure 6:**
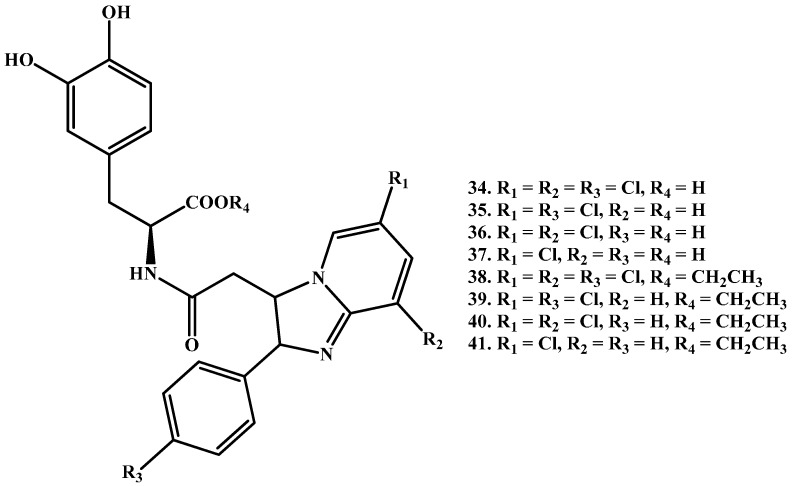
Chemical structures of substituted 2-phenyl-imidazopyridine-3-acetic acid amide prodrugs of LD.

Denora et al. [59] proposed a series of novel amide LD prodrugs (Figure 6, **34**–**41**), called Dopimid compounds. The series have high BBB penetrating potential due to substituted 2-phenyl-imidazopyridine-3-acetic acid. Of these Dopimid compounds, **34, 35, 38** and **39** were found to be stable against chemical hydrolysis, although they showed faster breakdown in diluted rat serum at 37 °C, and were able to penetrate the CNS. Moreover, a dose- and time-dependent elevation of DA levels in rat medial prefrontal cortex after intraperitoneal administration of compounds **38** and **39** was obtained. Similar results were also obtained by Eltayb et al. after i.p. administration of LD [60].

### 1.3. Dimeric Amide Prodrugs

**Figure 7:**
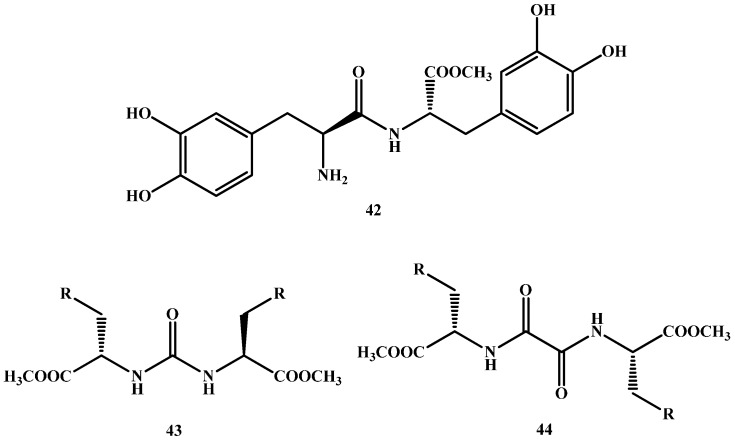

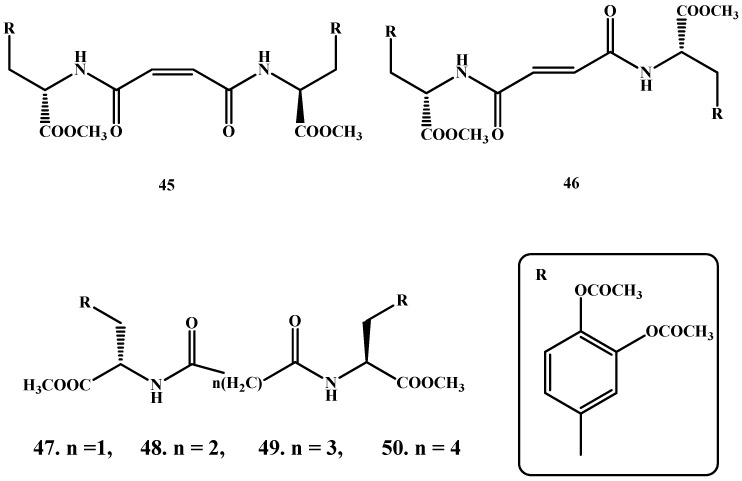
Chemical structures of dimeric amide prodrugs, **42**–**50**.

Dimeric prodrugs are, in essence, two identical molecules attached directly or indirectly using a spacer [61,62,63,64,65]. Felix and co-workers [66] have efficiently synthesized LD dimer prodrug without a spacer (**42** in Figure 7). On the other hand, Stefano and co-workers have synthesized various LD dimers [67,68,69,70] with different spacers used for each molecule (**43**–**50** in Figure 7). 

All dimeric prodrugs made have shown stability in buffer solution (pH 1.3 and pH 7.4), as well as a slow release of LD when tested in human plasma. Moreover, after oral administration of these compounds, the concentration of dopamine decreased much more slowly than that revealed with LD oral administration.

### 1.4. Carrier-Mediated Prodrugs

In this approach drugs are linked to an endogenous transporter substrate like amino acids, glucose, and other hexoses. This is aimed at the utilization of active transport mechanisms to ease BBB penetration, followed by bioconversion to yield the parent drug [35,71]. Most widely used transport systems in the prodrugs approach are glucose transporter (GLUT1), large neutral amino acid transporter (LAT1), monocarboxylic acid transporter (MCT) and peptide transport systems [72,73,74,75].

**Figure 8:**
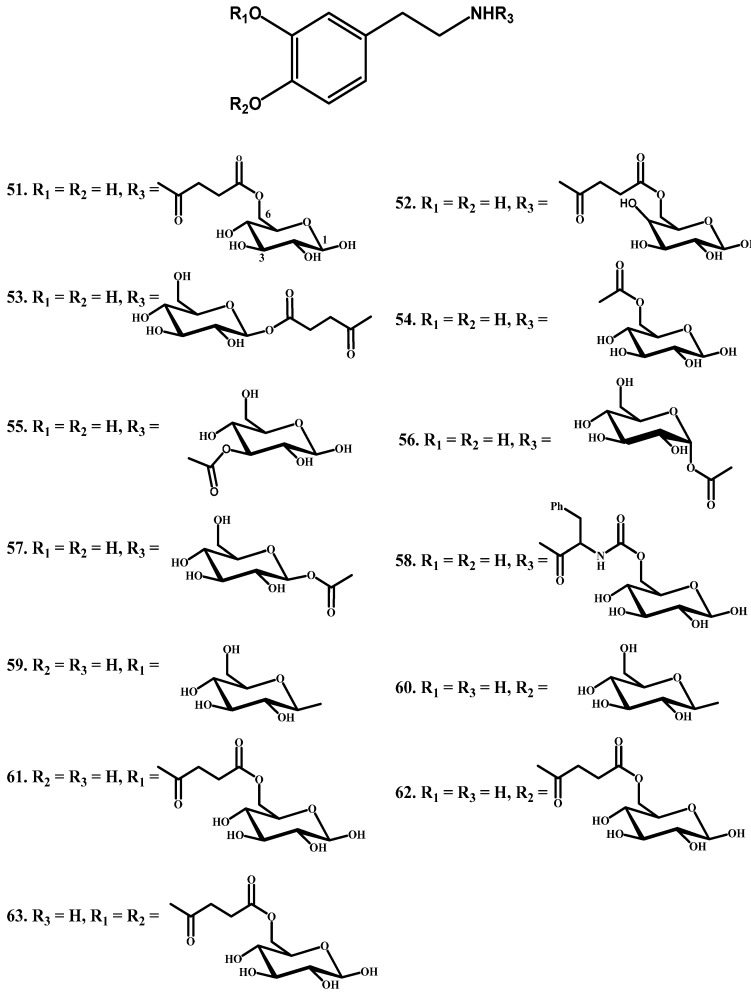
Glycosyl DA derivatives, **51**–**63**.

Novel DA glycosyl derivatives which can be transported by GLUT1 have been synthesized. Fernandez and co-workers [76,77] have synthesized glycosyl derivatives by attaching sugar and dopamine through a succinyl linker, carbamate bond, glyosidic, and ester bonds. They linked the amino group of DA to the C-6, C-3 and C-1 of the sugar through a succinyl linker (**51**–**53**, Figure 8) or a carbamate bond (compounds **54**–**56**). In another series, the sugar was linked to the phenolic groups of dopamine through a glyosidic bond (**59** and **60**) and ester (**61**–**63**) bonds, and then the affinity of these prodrugs for glucose carrier GLUT1 was tested using human erythrocytes. When DA-glycoconjugates were incubated with the brain extracts, the nature of the bond attaching DA with glucose affected the rate of DA release. The glycosyl conjugates substituted at the C-6 position of the sugar were more potent inhibitors of glucose transport in contrast to C-1 and C-3 substituted prodrugs. Among the studied compounds, the carbamate derivatives **52**, **56** and **57** were the prodrugs of choice, in particular compound **54**, which showed the best affinity for GLUT1, even higher than glucose itself.

**Figure 9:**
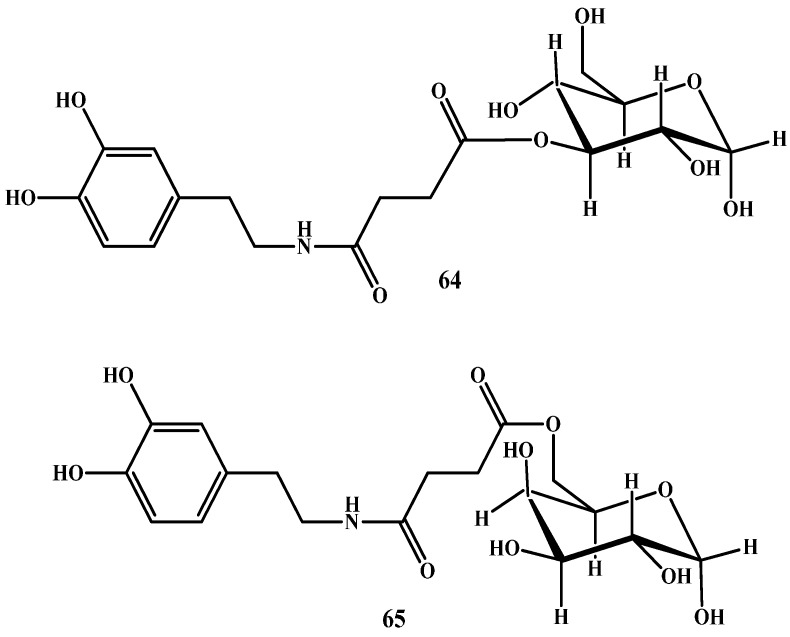
Chemical structures of glycosuccinyl-derivatives of DA, **64** and **65**.

In another study, Bonina et al. and Ruocco et al. [78,79] have prepared sugar-DA prodrugs in which DA was attached to the C-3 position of glucose (**64** in Figure 9) and to C-6 of galactose (**65** in Figure 9) by a succinyl spacer. Pharmacological studies showed that both prodrugs were more active than LD in reversing reserpine-induced hypolocomotion in rats. 

### 1.5. Peptide Transport-Mediated Prodrugs

**Figure 10:**
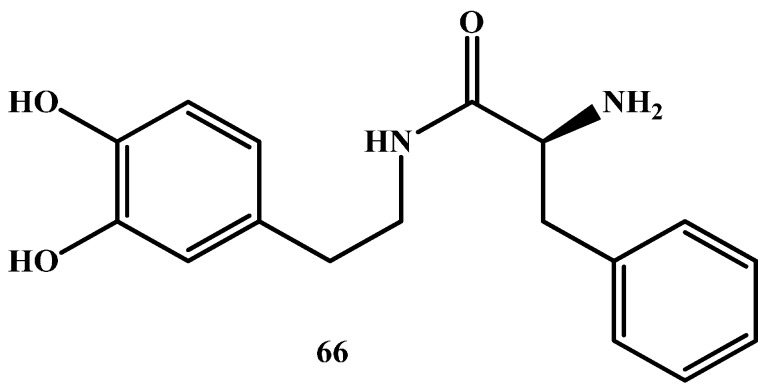
Structure of 2-amino-*N*-[2-(3,4-dihydroxy-phenyl)-ethyl]-3-phenylpropionamide (DA-PHEN), **66**.

Giannola et al. [80] have proposed a 2-amino-*N*-[2-(3,4-dihydroxyphenyl)-ethyl]-3-phenyl-propionamide dopamine prodrug (DA-PHEN) (Figure 10) [81]. It was synthesized by condensation of dopamine with a neutral amino acid to interact with the BBB endogenous transporters and readily enter the CNS. DA-PHEN undergoes slow cleavage by cerebral enzymes (t_½_ 460 min) and yields free dopamine in the brain, but it is rapidly hydrolyzed in human plasma (t_½_ 28 min). Chemical stability studies on DA-PHEN proved that no DA release happened in the gastrointestinal tract, also the prodrug can cross through a simulated intestinal mucosal membrane. 

Recently, De Caro et al. [81] studied in vitro the ability of DA-PHEN to penetrate the CNS. The team used in their study parallel artificial permeability assay (PAMPA) and Caco-2 models. Despite the relatively low molecular weight (300.35 Da) and the estimated experimental value [80] of log D_Ph 7.4_ (0.76) of DA-PHEN which indicates good potential for passage through biological membranes, they noticed very limited transport through PAMPA-BBB [81]. In fact, the apparent permeability was 3.2 × 10^7^ cm/s, indicating low capacity of DA-PHEN to penetrate BBB by passive transcellular route. Transport trials via Caco-2 cells showed marked increase of DA-PHEN flux with regard to that calculated in PAMPA-BBB system. However, high penetration rates seen in DA-PHEN cannot be obtained only by the simple diffusion, but may also involve carrier mediated transport [82].

**Figure 11:**
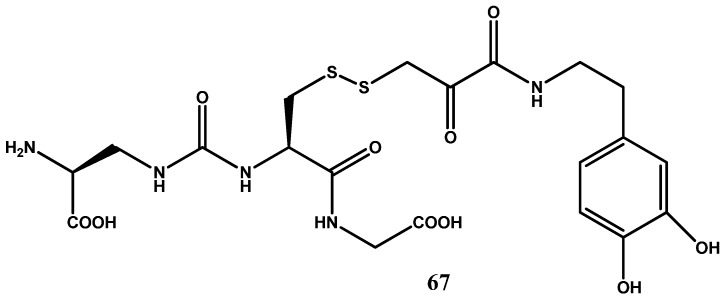
Glutathione conjugated prodrug of dopamine, **67**.

In another study, More and Vince [83] focused on the glutathione uptake transporters that are located on the luminal side of the BBB in attempt to increase BBB penetration of dopamine. The broad substrate specificity displayed by these transporters provides ample opportunity for rational prodrug design. The design of glutathione transporter targeted prodrug involved three components: the carrier, glutathione (GSH), the active drug, and a suitable linker for conjugation of the carrier with the drug molecule. The prodrug **67** (Figure 11) in which the dopamine is covalently linked via an amide bond to glutathione (GSH) demonstrated a high affinity for the GSH transporter at the BBB, liberated dopamine at the active site, and showed good stability balance between the periphery and brain.

### 1.6. Chemical Delivery Systems

**Scheme 2:**
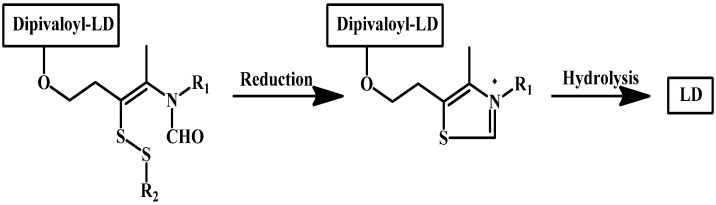
Activation mechanism of carboxylic acid-thiazolium prodrugs of LD.

**Figure 12:**
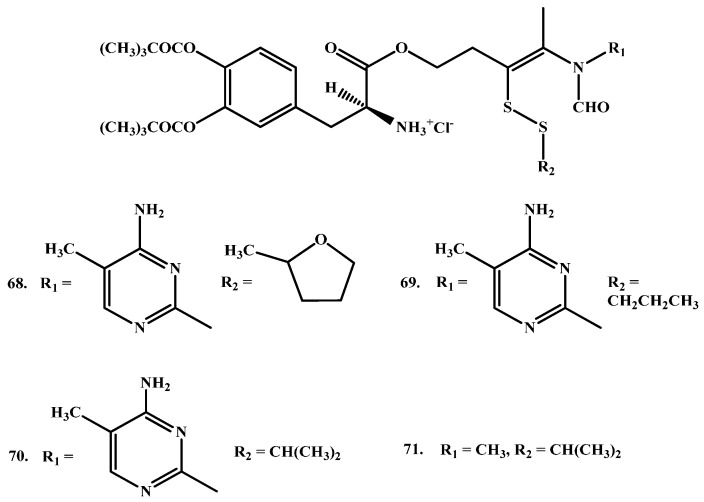
Chemical structures of carboxylic acid-thiazolium prodrugs of LD, **68**–**71**.

Ishikura and co-workers synthesized LD prodrugs by an esterification of LD carboxylic acid with a thiazolium moiety [84]. Four novel compounds were made (Figure 12). Ishikura and his team’s novel compounds rely on redox ring closure reactions of *cis*-2 formylaminoethenylthio derivatives to quaternary thiazolium derivatives and LD liberation after hydrolysis of the ester bond, as illustrated in (Scheme 2). The disposition of the prodrugs was evaluated by measuring the concentrations of DA regenerated after intravenous administration of the prodrugs and the results were compared with those for LD itself. The plasma levels of DA demonstrated no significant differences between DA and the prodrugs. In contrast, however, brain levels of DA were remarkably elevated following administration of the prodrugs. These findings suggest that a redox ring-closure system to a quaternary thiazolium can be used as an alternative chemical delivery system to the brain. 

### 1.7. Cyclic Prodrugs

**Figure 13:**
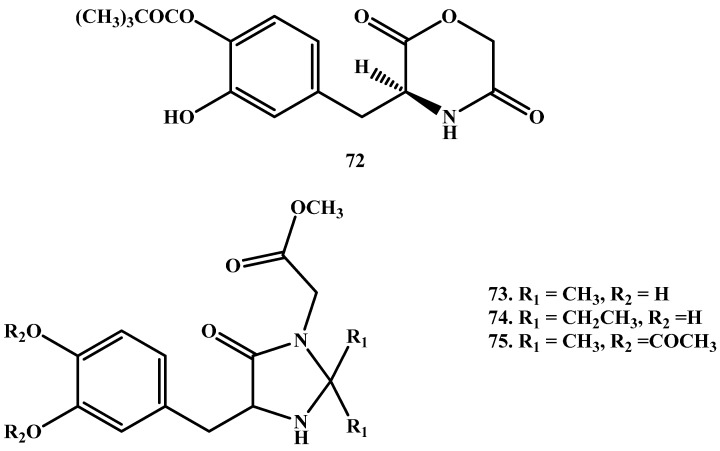
Chemical structures of cyclic prodrugs of LD, **73**–**75**.

Cingolani and co-workers [85] have prepared l-3-(3-hydroxy-4-pivaloyloxybenzyl)-2,5-diketomorpholine ring (Figure 13), to enhance the stability toward GI hydrolysis and to liberate LD in human plasma after enzyme-catalysed hydrolysis. Further, Giorgioni et al. [86,87] synthesized three cyclic compounds through the introduction of LD functional groups into animidazoline-4-one ring. The use of this ring particularly was based on its previously assured efficiency in protecting enkephalins from aminopeptidase degradation. The cyclic compounds were sufficiently stable in the acidic environment of the stomach; their absorption occurred in the intestine, and were capable to release LD to the brain in a slow manner.

### 1.8. Enzyme Model (Intramolecular Processes)

Despite some success that has been achieved using derivatives of DA and LD to supply DAin adequate concentrations and sustained release manner, the prodrugs chemical approach requiring enzyme catalysis has many limitations including many intrinsic and extrinsic factors that can alter the process. For example, the efficiency of many prodrug-activating enzymes may be different according to age-related physiological changes, drug interactions, or genetic polymorphisms, causing variation in clinical activity. Therefore, new derivatives for treatment of PD with drugs having higher bioavailability than the existing medications, and with the ability to liberate dopamine in a sustained fashion through chemical conversion without a need for enzyme catalysis is crucially needed [88].

In the past ten years, Karaman’s group have utilized molecular orbital methods such as ab initio and density functional theory (DFT) for the design of a large number of prodrugs which have the potential to undergo intramolecular cleavage to furnish the parent drugs and non-toxic linkers. The cleavage inintramolecular chemical rate is solely dependent on the nature of the linker and the physiologic environment to which the prodrug is exposed. 

**Figure 14:**
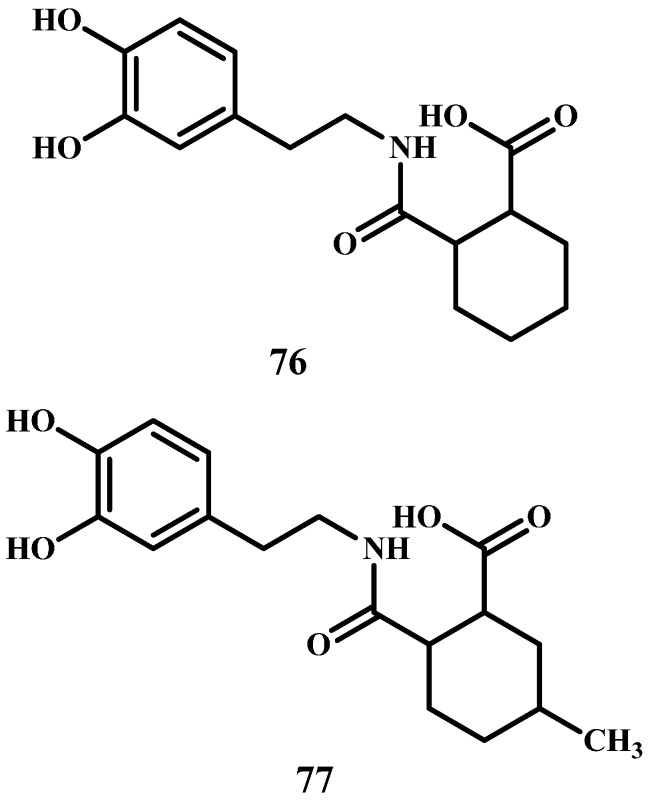
Chemical structures of DA prodrugs **76** and **77**.

Among others, they designed and synthesized a number of DA prodrugs to be used in the treatment of Parkinson’s disease with higher bioavailability than the existing medications. The synthesized prodrugs have moderate hydrophilic lipophilic balancewhich is important for their absorption and distribution [89], are soluble in physiological environment, their linkers are non-toxic, and have the potential to release DA in a sustained release manner (**76**–**77** in Figure 14). Kinetics study on **76** revealed that the prodrug was intraconverted to DA with t_½_ values of 60.3, 54.66, 99.93 and 138.13 h in 0.1 N HCl, buffer pH 2.2, buffer pH 5.5 and buffer pH 7.4, respectively. On the other hand, prodrug **77** was readily converted in 0.1 N HCl, buffer pH 2.2, pH 5.5 and pH 7.4 with t_½_ values of 48.34, 54.22, 131.98 and 193.42 h, respectively. The in silico and in vivo (rat) results demonstrated that both prodrugs are tolerable with low toxicity.

In vivo pharmacokinetics study is underway and it is believed that an oral enteric coated dosage form (to avoid conversion in acidic medium) will give pharmacokinetics profile with improved bioavailability than the current marketed drug [83,84,85,86].

## 2. Summary and Conclusions

Till today, PD treatments were based mostly on exogenous dopamine substitution within the striatum and LD is still the drug of choice to manage symptoms of PD. However, long-term therapy with l-dopa is associated with significant side effects. The main challenge in improving LD therapy is to reduce or remove the motor complications created or aggravated by noticeable LD plasma circulating level fluctuations. 

Dopamine and LD provide several sites in their chemical structures where it is possible to perform chemical alteration, this helped to have derivatives with enhanced physicochemical characteristics. In the past few years, researchers have conveyed their awareness towards designing targeted dopamine and LD prodrugs to replace exciting marketed drugs which have poor physicochemical and pharmacological properties. As mentioned above, dopamine and LD prodrugs should be soluble in both water and lipid, fully absorbed by gastrointestinal tract without any chemical degradation or metabolism, and have the ability to cross the BBB and hence to produce dopamine at the brain at consistent therapeutic level. Furthermore, an ideal prodrug should have sufficient and constant plasmatic levels to be able to provide continuous dopaminergic stimulation, thus preventing the manifestation and severity of LD-induced motor fluctuations and dyskinesia. 

There is promising data regarding using prodrugs for the treatment of PD. For example, ester prodrugs of LD have demonstrated to be suitable for intranasal delivery of LD [42,90]. This approach is considered as one of the administration routes that appear to enable the absorption of therapeutic agents to the brain bypassing the limitations of the BBB; most of the amide, cyclic, peptidyl or chemical delivery systems of DA prodrugs haveshown decreased enzymatic and hydrolytic susceptibility together with enhanced pharmacokinetic properties.

A promising and interesting approach suggested by Karaman’s group to overcome the limitation of the existing targeting prodrugs is model in which a parent drug is connected to enzyme model (intramolecular chemical device) and when the prodrug reaches the blood circulation within CNS, it undergoes intramolecular chemical conversion—without any regiment of enzyme allowing the active parent drug to be released in a sustained manner. The conversion rate of the prodrug to its parent drug is solely determined on the chemical features of the linker (enzyme model). This approach has the potential to, provide effective drug delivery system having selective targeting of DA and LD prodrugs to the striatum.

## Abbreviations

PD	Parkinson’s disease
DA	Dopamine
LD	LevoDopa
BBB	Blood brain barrier
CNS	Central nervous system
AUC	Area under the curve
6-OHDA	6-hydroxydopamine
i.p.	Intraperitoneal
COMT	Catechol-*O*-methyltransferase
DDC	Dopa-decarboxylase
MAO	Mono amino oxidase
GSH	Glutathione
DA-PHEN	2-Amino-*N*-[2-(3,4-dihydroxy-phenyl)-ethyl]-3-phenyl-propionamide
PAMPA	parallel artificial permeability assay
GLUT1	glucose transporter
MCT	monocarboxylic acid transporter
PDDP	dopamine-3-(dimethylamino) propanamide
LAT1	large neutral amino acid transporter
